# PHEWAS, TWAS, Mendelian Randomization in Osteoporosis Research: the good, the bad, and the ugly

**DOI:** 10.1007/s11914-026-00962-5

**Published:** 2026-04-10

**Authors:** Siwen Li, Katerina Trajanoska

**Affiliations:** 1 Department of Internal Medicine, Erasmus Medical Center Rotterdam, Rotterdam, The Netherlands; 2 Erasmus Medical Center, Dr. Molewaterplein 40, Rotterdam, 3015 GD Netherlands

## Abstract

**Purpose of review:**

While GWAS has identified many loci associated with bone mineral density (BMD), translating these findings into functional insights and treatments remains challenging. Post-GWAS methods such as Transcriptome-Wide Association Study (TWAS), Phenome-Wide Association Study (PheWAS), and Mendelian Randomization (MR) provide complementary strategies to prioritize genes and causal risk factors. This review summarizes findings from these studies.

**Recent Findings:**

TWASs have identified many potential causal genes for BMD, but only a few, such as PPP6R3, have been confirmed through functional validation. Several MR studies have provided increasing evidence of causal relationships between inflammatory bowel disease, NAFLD, COPD, and lower BMD, along with a higher risk of osteoporosis. PheWAS and MR also identify bone marrow fat as a risk factor for decreased BMD.

**Summary:**

It is essential to bridge the critical gap between statistical discovery and biological validation. Moreover, the lack of bone-specific transcriptomic data remains a significant limitation, underscoring the need to generate such datasets. At the same time, all MR evidence should be corroborated with other sources to strengthen causal conclusions.

**Supplementary Information:**

The online version contains supplementary material available at 10.1007/s11914-026-00962-5.

## Introduction

Osteoporosis represents a major public health concern, primarily due to its serious consequences, particularly fractures. Each year in Europe, osteoporosis accounts for 3 million fractures, leading to 2 million disability-adjusted life years and incurring direct costs of up to 36 billion euros [1]. Recent global projections estimate that the total number of hip fractures will almost double by 2050 compared to 2018 [2]. Therefore, preventing fractures remains a primary public health concern.

A complex interplay of genetic and environmental factors shapes the risk of osteoporosis and fractures. While some clinical risk factors are included in risk prediction models, such as the FRAX algorithm, these models do not account for all cases of fragility fractures [3]. A positive family history of osteoporosis significantly increases fracture risk [4], highlighting the important link between genetics and disease susceptibility. Clinically, osteoporosis is defined by its endophenotype, bone mineral density (BMD), which is also highly heritable (50–80%) [5]. Genome-wide association studies (GWASs) have discovered thousands of loci associated with BMD variation and osteoporotic fracture risk in the general population [6, 7]. Consequently, these large-scale GWAS studies have paved the way for several post-GWAS analyses to help us establish stronger causal links to genes or risk factors that cause disease. These analyses included high-throughput approaches, such as transcriptome-wide association studies (TWAS), phenome-wide association studies (PheWAS), and Mendelian randomization (MR). All three approaches leverage genetic variants to study relationships between genetic variants, gene expression, and phenotypes. Each of these methodologies provides unique insights into disease etiology, but their implementation comes with specific challenges (**Box 1**). TWAS integrates genetic and transcriptomic data to identify genes whose expression levels are associated with traits/diseases, offering mechanistic insights into gene regulation, which can then be further tested for causality using MR [8]. PheWAS scans genetic variants against a wide array of phenotypes and can help discover unexpected phenotypic effects of genetic variants used in MR and TWAS, providing insights into pleiotropy [9]. Finally, MR uses genetic variants as instrumental variables to infer causal relationships, addressing limitations of observational studies [10]. Together, these approaches provide a multi-layered view of the genetic basis of diseases, bridging molecular mechanisms and clinical outcomes.

**Box 1** | Limitations and challenges of PheWAS, TWAS, and MR.

**Table:** 

**TWAS ** **Tissue and Reference Panel Biases:** It is challenging to select the appropriate tissue and account for its unique expression profile. The accuracy of imputed gene expression relies on high-quality and representative reference panels. **Co-regulation and Multiple Hits per Locus: ** Due to linkage disequilibrium and shared expression quantitative trait loci, several genes in the same region can emerge as significantly associated, even if only one is truly causal. **Prediction accuracy is affected by heritability: ** The predictive accuracy of the genotype-expression model and the expression heritability significantly impact the power of TWAS. **Misinterpretation of Causality: ** TWAS does not test causal associations.	
**PheWAS** **Multiple Comparison Burden: ** A high number of phenotypes and a lack of independence among phenotypes. **Variable Quality of Phenotypic Data: ** Variability in phenotype definitions and misclassification may dilute associations. **Limited Power for Rare Phenotypes:** Many phenotypes have low case numbers and sometimes is also not possible to account for confounders.	
**MR ** **Instrument Validity:** Instruments may be weak or exhibit pleiotropy, potentially biasing causal estimates. **Canalization: ** Canalization introduces an additional layer of complexity by potentially weakening the genotype-exposure relationship, underestimating causal effects, and introducing variability across individuals. **Population Stratification and Generalizability:** Genetic variants can differ in frequency or effect size across ancestral groups. If not accounted for, these differences can create spurious associations or limit the generalizability. **Measurement Error/Reverse Causation:** Accurate measurement of exposures and outcomes is crucial to avoid misleading conclusions.	

In this review, we synthesize findings from the past 1–3 years using these approaches, highlighting their implications for osteoporosis research.

## Identifying Candidate Causal Genes Using TWAS

**Fig. 1:**
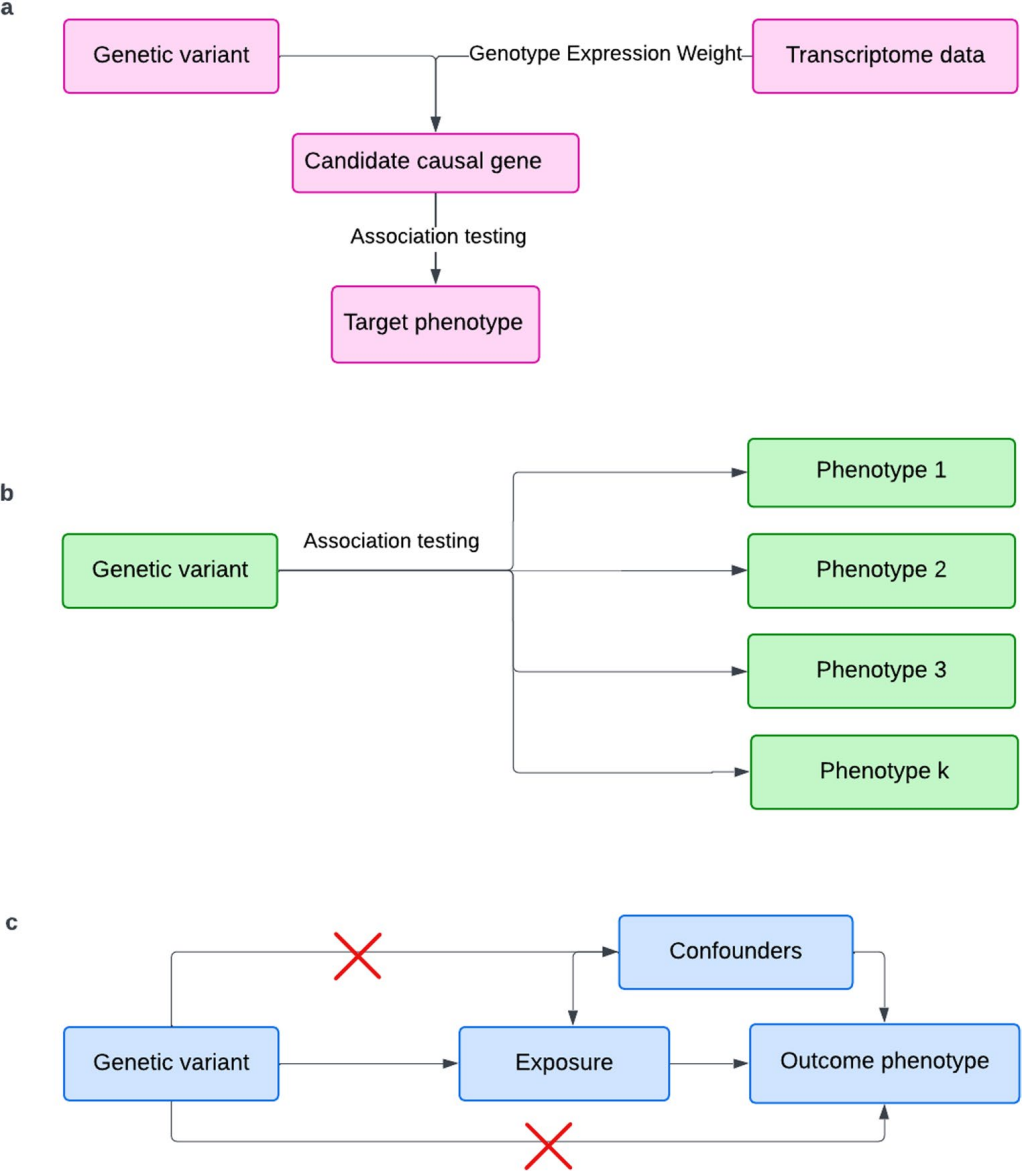
Schematics of transcriptome-wide association study (TWAS) [a], phenome-wide association study (PheWAS) [b], and Mendelian randomization study (MR) [c]

The TWAS approach utilizes expression quantitative trait loci (eQTLs) to pinpoint potential causal genes identified by GWAS, linking genetic regulation of gene expression to phenotypic outcomes (Fig. 1a). Gamazon et al. introduced this approach ten years ago [11]. Mai et al. [8] recently reviewed the advancements and applications of TWAS and provided a comprehensive overview of the technical aspects of the method and the available resources. The TWAS analyses have been significantly advanced by the development and availability of high-quality eQTL datasets from projects such as the Genotype-Tissue Expression (GTEx) project [12], the eQTLGen consortium [13], the Brain eQTL Almanac (Braineac) [14], PsychENCODE [15], the database of immune cell expression, expression quantitative trait loci and epigenomics (DICE) [16], and other eQTL resources [17]. A common limitation shared by all eQTL databases is the lack of bone-related transcriptomic datasets. Below, we summarize the latest TWAS findings in bone research.

Al-Barghouthi et al. [18] have performed a large scan using the GTEx eQTL reference dataset across 49 tissues to conduct TWAS and eQTL colocalization analyses on the largest GWAS on heel estimated BMD, and have identified 2,156 protein-coding genes, out of which 512 were putatively causal protein-coding genes. The significant associations spanned across all 49 tested tissues, with the highest number of colocalized SNPs in cultured fibroblasts, subcutaneous adipose tissue, and tibial artery and nerve tissues. Additionally, 13% (*n* = 67) of the predicted causal genes were previously identified as bone-associated genes. This gene set was generated using a curated Gene Ontology database combined with the Human-Mouse: Disease Connection database, focusing on genes involved in bone-related biological processes (*N* = 1,399). A proportion of these genes were enriched for ontologies related to bone ossification, skeletal development, and osteoblast differentiation, among others. Notably, 142 genes had available knockout models in the International Mouse Phenotype Consortium (IMPC), and up to 6.5% of the knockouts had alterations in BMD. Among the 512 putatively causal protein-coding genes, *PPP6R3* was prioritized for functional follow-up. The Ppp6r3-depleted mice had lower BMD, deteriorated bone microarchitecture, and increased bone turnover, prioritizing this gene as a putative causal gene for BMD, i.e., osteoporosis.

In a similar effort, Liu et al. [19] explored total body BMD and any-type of fracture. In the TWAS analysis, the authors identified 148 genes significantly associated with TB-BMD (*n* = 66,628), of which 52 were replicated in-silico in an independent dataset using femoral neck and lumbar spine BMD (*n* = 7,697). In addition, 38 genes were associated with any-type of fracture, of which 31 replicated. A follow-up Summarized Mendelian Randomization (SMR) analysis revealed that 78 of the 88 significant genes may have potential causal effects on TB-BMD or fracture in at least one tissue. Among these, 64 genes have been identified in previous GWASs or TWASs related to osteoporosis, including *ING3*,* CPED1*, and *WNT16*, as well as 14 novel genes, such as *DBF4B*,* GRN*,* TMUB2*, and *UNC93B1*. Nevertheless, it is worth acknowledging the sample overlap between the discovery and validation datasets in the fracture analysis, which can overestimate the proportion of replicated genes. On the other hand, Liu et al. [20] identified 70 significant genes associated with volumetric BMD, including previously identified osteoporosis-related genes such as *LYRM2* and *NME8*, as well as some novel loci such as *DNAAF2* and *SPAG16*. Next, Zhu et al. [21] identified 204 genes significantly associated with heel estimated BMD. Among these, 144 genes have been previously associated with bone-related phenotypes (evidence from multiple sources, including the literature, protein-protein interaction networks, and pathway analysis), and 50 genes have not been previously associated with any bone-related phenotype. Xu et al. [22] performed TWAS, focusing on skeletal muscle, peripheral-, and whole-blood gene expression, as well as total body BMD and lean mass as outcomes in children. For the BMD analysis, they identified 174 genes, specifically highlighting *IKZF1* and *HKB*. Finally, Su et al. [23] conducted in-silico TWAS analyses [24] and identified 21 structural variants, most of which mapped to genes not previously associated with bone phenotypes.

A key limitation of the last five efforts is the lack of replication and independent validation. Specifically, these efforts did not include functional follow-up of the identified genes to directly test their roles in bone biology, which restricts the ability to draw further conclusions or refine the list of candidate causal genes.

Abood et al. [25] examined the impact of noncoding transcripts as potential causal genes, with a specific focus on long noncoding RNAs. The authors performed a combined analysis of allelic imbalance in human acetabular bone fragments with TWAS and eQTL colocalization analysis using data from GTEx, discovering 333 significant lncRNA-BMD associations. However, only 14.4% (*n* = 48) colocalized with eQTL in at least one GTEx tissue, and only 31 lncRNAs (< 1%) were significant in both the TWAS and eQTL colocalization analyses. The effects of structural variants and lncRNAs on bone phenotypes are still not well understood.

Although many eQTLs are shared across tissues, the lack of eQTL data specific to bone and bone cells can lead to potential effector genes with bone-specific eQTLs being overlooked. A notable advancement in the field is the work by Mullin et al. [26], who developed a human osteoclast-specific eQTL resource and identified candidate GWAS effector genes linked to multiple bone phenotypes, including estimated heel BMD, skull BMD, and Paget’s disease. In recent work, Mullin et al. [27] conducted eQTL colocalization and SMR using gene expression data from osteoclast-like cells and prioritized several estimated heel BMD genes, drawing on the largest GWAS on estimated heel BMD. However, only 38% of the colocalizing eQTLs and 19% of the identified SMR genes overlapped with the 512 prioritized genes by Al-Barghouthi et al. [18] (discussed above), highlighting the need to incorporate bone-specific eQTLs in future research.

Currently, several comprehensive online databases exist for in-silico lookup of previously reported TWAS associations. The TWAS-hub (http://twas-hub.org/) provides summary statistics for 75,951 gene-trait associations across 342 traits. The webTWAS (http://www.webtwas.net/) collects 276,868 gene-trait associations from 1,394 fine-mappable GWAS summary statistics. Lastly, the TWAS Atlas (https://ngdc.cncb.ac.cn/twas/) stores high-quality TWAS statistics with 401,266 gene-trait associations across 257 traits and 135 tissues, with results archived per publication as of 2022. Although all these websites include information on bone phenotypes as tested outcomes, they again lack data on bone cells and tissues; therefore, the interpretation of results should consider this limitation.

### Identifying Unexpected Gene Associations Using PheWAS

PheWAS tests the association between a single genetic variant or a genetic risk score (GRS) and a wide range of clinical phenotypes (Fig. 1b). This is a relatively new approach, with the first PheWAS study serving as a proof-of-concept published in 2010 [28]. An initial success of PheWAS in the osteoporosis field was reported in 2016 when Wang et al. [29] identified a missense variant (rs113396273) in the Collagen Type 6 Alpha 5 chain (*COL6A5*) associated with osteopenia and other bone and cartilage disorders in European individuals (*n* = 29,722). Interestingly, the variants chosen for the PheWAS analysis were first identified in a PheWAS conducted on a murine cohort (*n* = 150) of recombinant inbred strains (the BXD family), where 3,805 genotypes were initially associated with up to 4,230 traits and 602,746 endophenotypic traits across 16 tissues. This is the first study to combine mouse and human PheWAS cohorts to validate and translate key genome-to-phenome relationships.

Currently, up to 4,000 PheWAS studies have been performed, with more than half only in the past three years. Over the last three years, only a handful of studies have used osteoporosis-related phenotypes in their PheWAS analyses, providing limited insight into unexpected risk factors influencing these phenotypes. For instance, a PheWAS study using GRS for age at natural menopause [30] reported an association with LS-BMD among other traits. Similarly, a PheWAS scan using GRS for body mass index (BMI) [31] has also been associated with BMD. However, this is not novel information, as both are known risk factors for bone outcomes. A study by Yuan et al. revealed that genetic liability to Coeliac disease was significantly associated with a higher risk of osteoporosis [32]. A PheWAS scan in the Taiwan Precision Medicine Initiative has evaluated pathogenic variants mapping to genes implicated in Non-Syndromic Hearing Loss and has reported associations with wrist fracture (GJB2 p.V37I) [33] and fractures of the lower limb (KCNQ4 c.546 C > G) [34]. However, further research and validation efforts are needed to elucidate the underlying mechanisms. The PheWAS approach can be effectively integrated with MR to assess the causal relationships underlying PheWAS-identified associations. In a compelling work, Xu et al. [35] used GWAS data on bone marrow fat fraction (BMFF) measured in the femoral head, total hip, femoral diaphysis, and spine from over 48,000 UK Biobank participants to develop a polygenic risk score. This score was then used in a PheWAS examining up to 15,000 phenotypes across 17 disease categories. The analysis revealed significant associations between the BMFF polygenic risk score and osteoporosis and fractures. In the downstream analysis, MR further validated these findings, indicating that increased marrow adiposity at the diaphysis and total hip is causally linked to osteoporosis.

## Identifying causal risk factors using MR

MR has undeniably emerged as a powerful tool for addressing causal questions in observational research (Fig. 1c); however, the abundance of MR studies over the past three years varies in quality and relevance, hindering clear interpretation and limiting the applicability of current MR findings. This is a common concern across various fields, and the field of osteoporosis research is no exception. Recently, Burgess et al. [36] provided a comprehensive review of the common pitfalls in conducting a reliable MR analysis. The first highlighted key issue is evaluating inappropriate research questions (“Is the research question addressable using MR?”). It’s important to have a strong logical rationale for why a research question warrants further exploration. The initial MR studies have made significant strides toward clarifying whether the associations observed in cross-sectional studies are truly causal or confounded by other variables [37]. One significant advancement in the field was the development of two-sample MR, which proved highly beneficial for studying associations between risk factors and outcomes in settings where both the exposure and outcome were not measured in the same study. Additionally, this approach helped improve statistical power. However, it also led to an increase in the number of studies, some of which are valuable but introduced certain challenges.

**Fig. 2:**
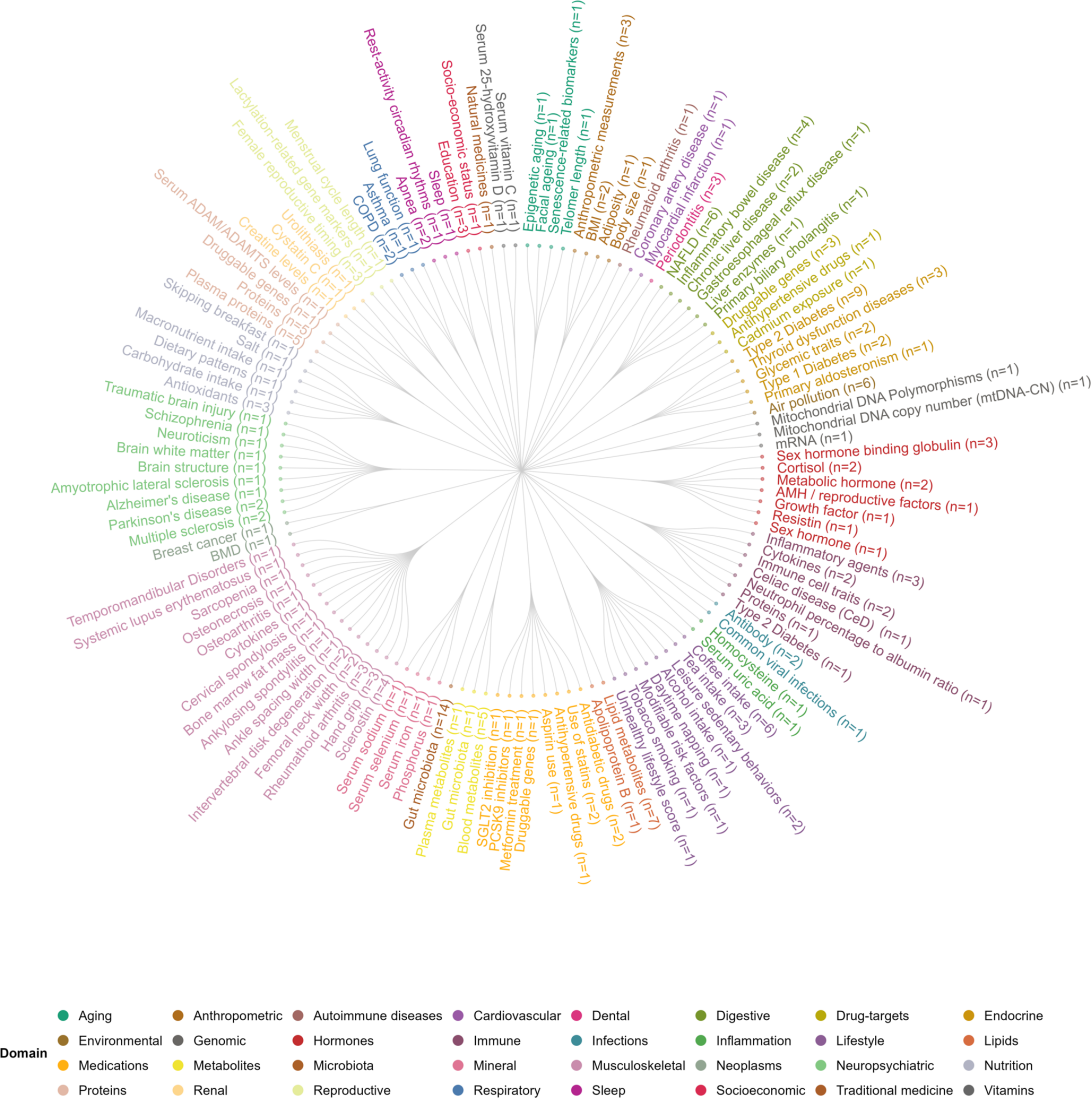
Summary of 32 risk factor domains tested for associations with bone outcomes. n: number of studies that have evaluated the particular trait as exposure for MR analysis using bone outcomes; COPD: chronic obstructive pulmonary disease; NAFLD: non-alcoholic fatty liver disease; GERD: gastroesophageal reflux disease; FGF: fibroblast growth factor; sRANKL: soluble receptor activator of NF-κB ligand; OPG: osteoprotegerin; BMI: body mass index; WHR: waist-to-hip ratio; WC: waist circumference; HC: hip circumference

Over the past three years, up to 265 MR studies have been conducted in the bone field, out of which 229 studies focused on osteoporosis (*n* = 117), fracture risk (*n* = 39), and BMD (*n* = 164) as main outcomes, covering risk factors across 32 domains (Fig. 2, Supplementary Table 1). 32 of these studies (*n* = 229) perform bidirectional MR, in which bone phenotypes serve as both exposures and outcomes, typically used when the direction of effect is uncertain (Supplementary Table 2). The remaining 29 studies studied BMD as the main exposure for various outcomes (Supplementary Table 3).

### Chronic Conditions and bone Outcomes

A chronic condition that has been extensively evaluated in the last few years is non-alcoholic fatty liver disease (NAFLD). Zhou et al. reported a causal association between genetically predicted NAFLD and osteoporosis [38]. Similarly, Pei et al. [39] identified a causal relationship between NAFLD and femoral neck BMD (FN-BMD), along with a suggestive association between NAFLD and osteoporosis, which was also supported by findings from Cui et al. [40]. In addition, a causal link between NAFLD and forearm BMD and total body BMD has been reported [41, 42]. In contrast, Huang et al. found no significant effects of NAFLD on BMD in four skeletal sites (total body, femoral neck, lumbar spine, forearm) [43]. When Pei et al. [39] used the same data source (FinnGen) to derive SNPs for the NAFLD instrumental variable as Huang et al. [43], they similarly reported a null effect on BMD. This highlights the importance of carefully considering differences in instrumental variable construction when comparing findings across studies. A causal relationship between Crohn’s disease [44], inflammatory bowel disease [44–47], ulcerative colitis [44], and osteoporosis has also been reported by several MR studies.

Next, MR studies have also evaluated the role of COPD and Asthma in the development of osteoporosis [48, 49]. Osteoporosis and vertebral fractures are quite common in patients with advanced COPD and show a significant relationship to the mortality of these patients [50, 51]. A causal relationship between COPD and the risk of osteoporosis has been reported by Dou et al. [52], along with evidence that COPD is negatively associated with heel BMD [48] but no other site-specific BMDs. However, findings from Yang et al. did not support a causal relationship between COPD and heel BMD [49]. The discrepancy between the two studies likely arises from the differences in the construction of the instrumental variable.

Furthermore, MR has reported that both childhood-onset asthma and adult-onset asthma [53] may have a causal effect on osteoporosis, with a higher risk of osteoporosis observed in adult-onset asthma. Nevertheless, the results need to be interpreted with caution, as the effect estimate was not robust across different MR estimators. MR, like any other statistical approach, is subject to limitations. To address potential biases and strengthen causal inference, it is common practice to test a range of MR estimators, including inverse variance weighting (IVW), the weighted median, and mode-based estimators, to evaluate whether the causal effect estimates are consistent across methods that rely on different assumptions about the validity of the instrumental variable.

### Medications and Bone Outcomes

An MR study has suggested that genetically predicted calcium channel blockers (CCBs) may increase fracture risk, whereas angiotensin receptor blockers (ARBs) may decrease fracture risk [54]. No effect was reported for alpha-blockers, angiotensin-converting enzyme inhibitors (ACEIs), beta-blockers (BBs), loop diuretics, potassium-sparing diuretics (PSDs), and thiazide diuretics. The findings on ARBs, broadly classified as Renin-angiotensin-aldosterone system (RAAS) inhibitors, are particularly interesting, as animal studies have demonstrated increased bone mass and strength through the blockade of angiotensin II pathways [55]. Nevertheless, similar effects have not been observed with other classes of RAAS inhibitors, specifically the angiotensin-converting enzyme (ACE) inhibitors.

In similar study settings, two MR studies have reported conflicting effects of PCSK9 inhibitors on osteoporosis risk. These inhibitors are monoclonal antibodies that decrease LDL cholesterol by blocking PCSK9, a protein that typically promotes the breakdown of LDL receptors. PCSK9 inhibitors play a key role in managing cardiovascular disease, and it is hypothesized that they could affect bone metabolism by lowering LDL levels [56]. However, evidence from these two MR studies remains inconsistent: one two-sample MR study reported a lower osteoporosis risk [57], whereas another reported an increased osteoporosis risk [58]. One difference between the studies lies in the SNPs used to construct the instrumental variable (assuming there are no other technical biases), which brings us to the second key consideration in evaluating the credibility of an MR study: “*Are the chosen genetic variants appropriate*?” [36]. MR analysis has also suggested a potentially beneficial effect of another class of lipid-lowering medication, namely the peroxisome proliferator-activated receptor (PPARs) agonists (bezafibrate and fenofibric acid) [59]. The drug-target MR approach analysis has also identified potential novel drug targets, suggesting that ANGPTL3 and APOC3 may serve as new non-statin lipid-lowering drugs for treating or preventing osteoporosis.

### Omics and Bone Outcomes

Advancements in proteomics have significantly transformed the MR field, enabling the identification of potential causal proteins and druggable targets for diseases, including osteoporosis. Up to 13 studies have used proteomics as an exposure, with 11 performing protein-wide association analyses [60–72], and one has focused on serum ADAM/ADAMTS levels [60], and another on mTOR-dependent EIF-4E circulating protein levels [70]. The latter studies have shown ADAM/ADAMTS levels to have a suggestive impact on estimated heel BMD and no effect on femoral neck, lumbar spine, and forearm BMD, whereas one mTOR-dependent circulating protein influenced forearm BMD but not the other skeletal sites. The relevance of these proteins may be limited as the site-specific effects are unclear. One interesting study is that by Zhou et al. [71], where integrating exome sequencing findings with proteomics MR evidence has prioritized CD109 (cluster of differentiation 109) as a potential novel drug target for osteoporosis. The exome analysis revealed that heterozygous loss-of-function variants in CD109 are associated with increased BMD, and MR analysis further supported this, showing that lower circulating CD109 levels correlate with higher estimated heel BMD. Subsequent functional experiments confirmed that partial CD109 knockdown was associated with increased bone mineralization, reinforcing its therapeutic potential. Next, Michaelsson et al. [72] have identified 24 cardiometabolic proteins associated with fracture risk, of which SOST, NTproBNP, BNP, and CCDC80 have been demonstrated to have potential causal effects. Regarding CCDC80, the observational study and the MR analysis showed opposite effects, which the authors hypothesize could be due to horizontal pleiotropy. This study is a good example of how bringing together evidence from multiple, complementary study settings, so-called triangulation, can help us better understand the findings and strengthen the validity of MR findings.

Up to six studies [73–78] have been performed in the metabolomics domain in the last three years. A large-scale plasma metabolite study by Chen et al. [73] showed that orotate has an adverse effect on bone, as it is causally associated with lower estimated heel BMD and, in a separate independent cohort, with increased fracture risk. Next, Liu et al. [74] identified five metabolites that may contribute to osteopenia by combining SMR and colocalization analyses. Among these, biliverdin appeared to have a protective effect; however, the implications are unclear. Chen et al. [77] found 22, 10, 3, 7, and 2 metabolite links to estimated heel BMD, total body BMD, lumbar spine BMD, femoral neck BMD, and forearm BMD, respectively, with androsterone sulfate showing a strong effect across all site-specific BMDs. However, in this study, the authors used odds ratios to report continuous outcomes, which complicates the interpretation of the effect’s direction. Thus, authors need to report the risk estimates on the appropriate risk scale. Next, Gong et al. [76] report a causal association between the Adenosine 5’-monophosphate (AMP) to alanine ratio and BMD. However, the metabolite GWAS source cited for the metabolomics MR analysis does not include a measurement of the adenosine 5’-monophosphate (AMP) to alanine ratio, making it unclear or implausible to construct the instrumental variable. Moreover, this specific ratio is not commonly used in clinical or research practice, which further limits the interpretability of the findings. Therefore, caution is warranted when deriving conclusions from MR analyses if the construction of the instrumental variable is not clearly reported.

Finally, although it is less likely that SNPs associated with microbiome diversity could serve as a plausible exposure for MR [38], up to 15 MR studies have been conducted on microbiota [69, 78–91] in the past three years. Among protective taxa, *Coprococcus* (especially *Coprococcus2/3*) has consistently emerged, showing inverse associations with OP and positive associations with BMD across multiple independent cohorts (Chen et al. [88], Qiao et al. [80], Zhou et al. [81], Ma et al. [83], Wang et al. [89]). Similarly, *Prevotellacea* frequently demonstrated beneficial effects on BMD [86, 88], whereas *Burkholderiales* showed a negative association with OP and decreased osteoclast activation [83, 85, 87]. On the other hand, multiple *Ruminococcaceae* sub-groups have been implicated in decreased BMD and higher OP risk across studies (Chen et al. [88], Qiao et al. [80], Xue et al. [86], Wang et al. [89]). *Peptococcaceae* and *Desulfobacterota/Desulfovibrionaceae* were also repeatedly associated with BMD and osteoporosis [79, 88].

### Antiosteoporosis Medications and Non-Skeletal Outcomes

Romosozumab, a monoclonal antibody that targets sclerostin, is an effective treatment for osteoporosis in people at high fracture risk. However, two phase III randomized controlled trials [92, 93] have reported an increased incidence of adverse cardiovascular events in patients receiving romosozumab. In contrast, another randomized controlled trial did not observe this adverse effect [94]. An MR study [95] has shown that genetically lower sclerostin levels are associated with increased risks of hypertension, myocardial infarction, type 2 diabetes, and greater coronary artery calcification. However, a novel flexible machine learning MR approach found no effect of sclerostin on ischemic cardiovascular diseases [96]. Although the impact remains uncertain, drug agencies have been cautious and have issued contraindications for patients with a history of myocardial infarction or stroke.

## Validating genetic Association Through Functional Studies

While genetic approaches have transformed bone research, much of the subsequent work still lacks direct functional validation. Thus, the functional relevance of many identified genes and pathways remains unclear. Addressing this challenge requires systematic experimental validation in disease-relevant systems. Integrating genetic findings with in vitro cell models [97], gene editing screens [98], and in vivo knock-out or knock-in animal models [99, 100] can provide deeper insights into bone physiology and disease mechanisms. These approaches can facilitate the translation of genetic discoveries into mechanistic understanding and the development of potential therapies for bone disorders.

## Methodological Limitations

Techniques like TWAS, PheWAS, and MR are mainly statistical and depend heavily on assumptions and the quality of the input data. For example, TWAS relies on the accuracy of expression prediction models, MR depends on the quality of the GWAS data, and PheWAS depends on the quality and depth of the phenotypic data. Therefore, all results should be interpreted within the limitations of the methods (Box 1).

## Conclusions

Drug discovery is a lengthy, uncertain process, but prioritizing and advancing even a single target can significantly impact bone research. Human genetics provides a reliable way to identify and validate drug targets, with studies showing two to fivefold improvement in success rates. Recent TWAS studies have identified PPP6R3 as a potential causal gene for BMD, indicating that inhibiting it could increase BMD and serve as a possible therapeutic strategy for osteoporosis. Additionally, MR studies have identified several proteins with therapeutic potential, including CD109. While a wide range of risk factors and diseases have been examined in relation to bone outcomes, current MR findings do not reveal any novel discoveries beyond what is already known. In contrast, some other MR findings lack robustness and require further research. Furthermore, the increasing number of MR studies in the bone research field calls for careful evaluation of the findings, especially regarding the relevance of the research question and the construction of the instrumental variable. To ensure reliable MR findings, they should be interpreted using a triangulation framework that assesses the extent to which other types of studies provide support or challenge for the evidence of a causal relationship. When applied rigorously, MR can identify causal risk factors and drug targets with important clinical implications. 

## Supplementary Information

Below is the link to the electronic supplementary material.

Supplementary Material 1

## Data Availability

No datasets were generated or analysed during the current study.
